# Therapeutic peptides identification via kernel risk sensitive loss-based k-nearest neighbor model and multi-Laplacian regularization

**DOI:** 10.1093/bib/bbae534

**Published:** 2024-10-22

**Authors:** Wenyu Zhang, Yijie Ding, Leyi Wei, Xiaoyi Guo, Fengming Ni

**Affiliations:** Institute of Fundamental and Frontier Sciences, University of Electronic Science and Technology of China, No. 2006 Xiyuan Avenue, High tech Zone, Chengdu 610054, China; Yangtze Delta Region Institute (Quzhou), University of Electronic Science and Technology of China, No.1 Chengdian Road, Kecheng District, Quzhou 324000, China; Yangtze Delta Region Institute (Quzhou), University of Electronic Science and Technology of China, No.1 Chengdian Road, Kecheng District, Quzhou 324000, China; Macao Polytechnic University, Gomes Street, Macau Peninsula, Macau 999078, China; Yangtze Delta Region Institute (Quzhou), University of Electronic Science and Technology of China, No.1 Chengdian Road, Kecheng District, Quzhou 324000, China; Department of Gastroenterology, The First Hospital of Jilin University, No. 71 Xinmin Street, Chaoyang District, Changchun 130021, China

**Keywords:** therapeutic peptides, Laplacian regularized model, multi-information fusion, kernel risk-sensitive mean p-power error, sequence classification

## Abstract

Therapeutic peptides are therapeutic agents synthesized from natural amino acids, which can be used as carriers for precisely transporting drugs and can activate the immune system for preventing and treating various diseases. However, screening therapeutic peptides using biochemical assays is expensive, time-consuming, and limited by experimental conditions and biological samples, and there may be ethical considerations in the clinical stage. In contrast, screening therapeutic peptides using machine learning and computational methods is efficient, automated, and can accurately predict potential therapeutic peptides. In this study, a k-nearest neighbor model based on multi-Laplacian and kernel risk sensitive loss was proposed, which introduces a kernel risk loss function derived from the K-local hyperplane distance nearest neighbor model as well as combining the Laplacian regularization method to predict therapeutic peptides. The findings indicated that the suggested approach achieved satisfactory results and could effectively predict therapeutic peptide sequences.

## Introduction

Peptides constitute active substances, such as enzymes, hormones, antibodies, and interneurons, in the human body and can be directly absorbed and utilized by the human body [1]. Peptides can regulate the functional activities of cells and regulate and enhance the physiological functions and activities of the human body. Therapeutic peptides [2] have unique biochemical properties and are less immunogenic and less costly to produce. Cell-penetrating peptides (CPP) [3], known as Trojan horses, can naturally cross the lipid bilayer membrane, which protects cells from drug delivery and boosts the stimulation of immune cells. Quorum-sensing peptides (QSP) [4] can drive the group-sensing phenomenon, help establish communication between cells, coordinate gene expression, and regulate physiological activities. Recently, therapeutic peptides have attracted considerable attention as potential drug candidates and delivery vehicles. Researchers have attempted to efficiently produce synthetic peptides on a large scale. However, using biochemical assays to validate peptide properties and predict peptide types is both time-consuming as well as requiring significant manual effort. Screening and predicting therapeutic peptides using computational simulations followed by experimental validation has a higher efficiency and success rate than traditional bioassays [5–8].

Machine learning is an effective computational method for selecting predictive therapeutic peptides; peptide sequences are first converted to mathematical representations, followed by the application of machine learning models to discern underlying patterns within these features [9]. Machine learning algorithms can be combined with biological sequence features extracted from physicochemical properties [10–14] for therapeutic peptide prediction. Charoenkwan *et al.* [15] utilized SVMs and physicochemical properties data to develop an effective QSP predictor. Guo *et al.* [16] proposed PreTP-EL, which amalgamates various features with machine learning methodologies to identify diverse characteristics of therapeutic peptides. Wang *et al.* [17] measure the peptide similarity by considering the physicochemical attributes of amino acids, integrate various types of information using a novel MKL method, and finally utilize SVM for classification prediction.

Nowadays, machine learning algorithms have been combined with feature vectors obtained using deep learning feature extraction techniques for therapeutic peptide prediction [18–22]. The inACP computational model proposed by Zhong and Deng [23] combines deeply learned sequence features and protein structural features and integrates three machine learning classifiers to predict therapeutic peptides. Yan *et al.* [24] integrated sequence features from multi-perspective learning and tensor-based approaches to develop TPpred-ATMV, which could accurately predict several therapeutic peptides. Qiang *et al.* [25] developed CPPred to learn feature representations from random forest models from different perspectives and perform feature selection. Jiao *et al.* [26] proposed ATGPred-FL, which used a feature learning method and a two-step accuracy-based feature selection strategy combined with SVM to efficiently identify autophagy proteins.

However, the methods described above have some shortcomings. Some studies have not been evaluated on independent datasets to determine their predictive ability for unknown peptides. In contrast, this study conducted experiments on both the training set and the independent test set to evaluate both the model’s performance and its ability to predict unknown peptides. Some studies require further processing of peptide sequence features to conform to the input form of the model, whereas the proposed model directly predicts therapeutic peptides using feature vectors as inputs. Some existing therapeutic peptide prediction models only target one therapeutic peptide, whereas the method proposed can predict different types of therapeutic peptides by fine-tuning the model. This study proposes a classifier based on the kernel risk-sensitive loss-based k-nearest neighbor model, inspired by the K-local hyperplane distance nearest neighbor (HKNN) model, which calculates distance proximity [27] to construct local hyperplanes to classify different therapeutic peptides. The HKNN model provides a level of interpretability by leveraging the concept of nearest neighbors, which can be more intuitive to understand than some black-box machine learning models. To enhance the system’s resilience so as to significantly mitigate the impact of noise and outliers, the kernel risk-sensitive mean P-power error (KRP) [28] was embedded as a loss function into the cost function, and the prediction of therapeutic peptides was achieved by iteratively minimizing the cost function value. This study employs various similarity kernel methods for measuring the similarity of two samples and then uses Laplace mapping to maintain a high correlation between mutually similar samples, combined with Laplace regularization methods to improve model generalization. To efficiently integrate these similarity scores, multi-view learning [29] was used to estimate the score for each similarity and assign corresponding weights. The research contributions can be summarized as listed below.

1) The KRP was introduced into the HKNN model as a loss function that has upper and lower bounds that supersedes the original squared loss function. An objective function was constructed for each sample within the classes targeted for classification, aiming to enhance the model’s generalization capabilities. Due to the fact that the KRP derivative is smoother and eventually converges to 0, it is more resistant to noise.
2) Laplace mapping and regularization were introduced, combining multiple Laplace matrices, using multi-view learning to fully utilize the scores of different similarity methods, and employing a repetitive computational process to address the objective function.
3) The peptide sequences obtained from the pre-training of the embedded model using deep representation learning were used as feature vectors to better preserve the key information of the peptide sequences. The proposed model can directly predict therapeutic peptides using feature vectors as inputs instead of further processing of peptide sequence features to conform to the input form of the model.

## Materials and methods

### Therapeutic peptide sequence feature

**Figure 1:**
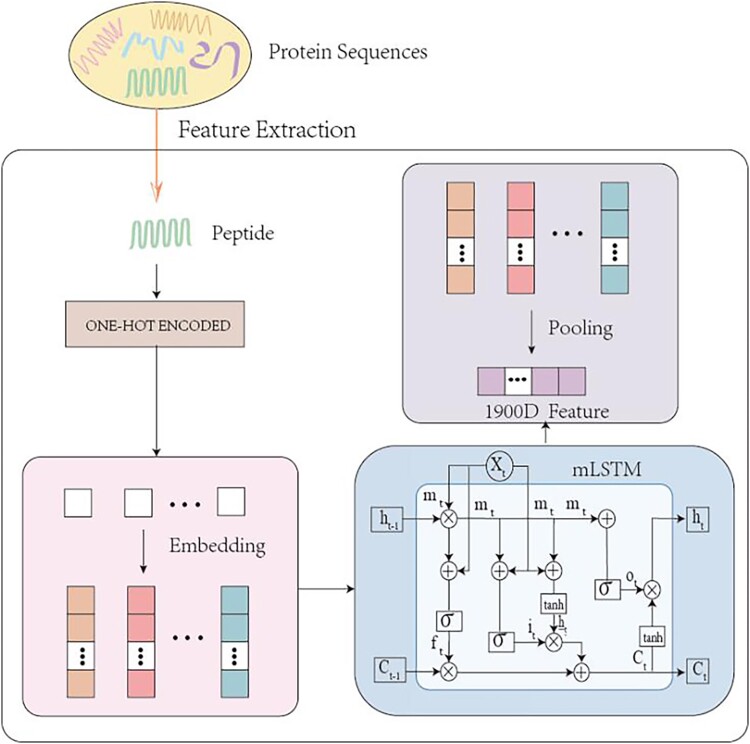
Process of the feature extraction.

In this paper, each peptide sequence corresponds to a 1900D fixed-length UniRep [30] vector, which captures the feature information in a sequence by performing a letter-by-letter prediction of the protein sequence. Each amino acid in the protein sequence is first represented as a vector using one-hot coding, which is sequentially inputted into LSTM, and a recursive structure is used to continuously pass the information forward to update its internal state. Finally an average pooling operation is performed to generate a 1900D feature vector for each peptide sequence. Figure 1 illustrates the UniRep feature extraction process. The UniRep feature captures rich information about protein sequences, including sequence patterns and evolutionary information, which facilitates more accurate protein property prediction and improves the model’s ability to generalize across proteins, allowing it to make effective predictions across different protein datasets [31].

### K-local hyperplane distance nearest neighbor algorithm

HKNN algorithm [32] synthesizes KNN and SVM algorithms to find the K points closest to the test sample in each class *c*, combines the weights of each of these points to construct a local hyperplane for each class *c*, calculates the distances between the test samples and the local hyperplane of each class, and categorizes the test samples into the class corresponding to the closest hyperplane c. KNN being the simplest machine learning method, which does not make any assumptions about the underlying data distribution. The feature space of the therapeutic peptides is often high-dimensional; KNN series methods have an advantage in capturing complex patterns in the data due to its reliance on the local structure of the data points. The formula for constructing a local hyperplane for class c is as follows:


(1) \begin{equation*} {LH}_c(X)=\left\{p\ |\ p=\sum_{n=1}^K{\alpha}_{c,n}{N}_{c,n},{\alpha}_{c,n}\in{R}^K,n=1,\dots, K\right\} \end{equation*}

where ${N}_{c,n}\in{\mathrm{R}}^{d\times 1}$ is the *n*th point in the training set of class *c* samples that is most adjacent to the instance, *d* is the dimension of the sample and ${\mathrm{\alpha}}_{c,n}$ is the weight of the *n*th point in class *c* samples. The formula for calculating the proximity of the instance $x\in{R}^{d\times 1}$ to the hyperplane of class *c* is given below


(2) \begin{equation*} S\left({\left({LH}_c(x)\right)}^2\right)={\lambda}_r{\left\Vert{\alpha}_{c,n}\right\Vert}^2+{\left\Vert x-\sum_{n=1}^K{\alpha}_{c,n}{N}_{c,n}\right\Vert}^2. \end{equation*}

Computing ${\mathrm{\alpha}}_{c,n}$ can be converted to solving the following linear system problem in matrix form:


(3) \begin{equation*} \left({N}_c^T\bullet{N}_c\right)\bullet{\alpha}_c={N}_C^T\bullet x \end{equation*}

where ${N}_c\in{R}^{d\times K}$ corresponds to the training set’s K feature vectors nearest to the test instance. ${\alpha}_c\in{R}^{K\times 1}$ consists of the weights of the K neighbors. The final test sample is categorized into the category *c* corresponding to the closest distance to the local hyperplane, and the formula is expressed as follows:


(4) \begin{equation*} {\mathrm{CLASS}}_c=\underset{\alpha_c}{\mathrm{argmin}}S\left({\left({LH}_c(x)\right)}^2\right). \end{equation*}

HKNN model combines local information from the nearest neighbors with a global perspective provided by the hyperplane distance. This could allow for a more nuanced understanding of the data, capturing both local patterns and broader trends. Moreover, HKNN considers local hyperplanes formed by neighboring points and is more resistant to noise and outliers than KNN models that rely only on nearest neighbors. The HKNN model has more hyperparameters, and both the number of neighbors and the hyperplane distance metric can be fine-tuned to be more adaptable to the prediction task of different therapeutic peptides.

### Kernel risk-sensitive mean p-power error

The KRP [33–35] represents a novel metric for similarity assessment founded on the nuclear risk-sensitive loss (KRL) [28], which can improve the robustness of the system by adjusting the parameter *p* to the proper value. KRP realizes a nonlinear mapping induced by the kernel that can transform the variables from the original feature space to the Gaussian kernel space to measure the differences between the variables. The Gaussian kernel produces smoother decision boundaries, has tunable bandwidth parameters that better fine-tune the model to the prediction task, and maintains superior performance in the face of noise [36]. The formula for measuring variables’ similarity by KRP is as follows:


(5) \begin{equation*} {L}_{\lambda, p}\left(A,B\right)=\frac{1}{\lambda}\int \exp \left\{\lambda{\left[1-{\kappa}_{\sigma}\left(A-B\right)\right]}^{\frac{p}{2}}\right\}d{F}_{AB}\left(a,b\right) \end{equation*}

where *p* is a power parameter greater than 0, ${\mathrm{\kappa}}_{\mathrm{\sigma}}\left(\bullet \right)$ represents a Gaussian kernel function with the following expression:


(6) \begin{equation*} {\kappa}_{\sigma}\left(a-b\right)=\exp \left(-\frac{{\left(a-b\right)}^2}{2{\sigma}^2}\right). \end{equation*}

**Figure 2:**
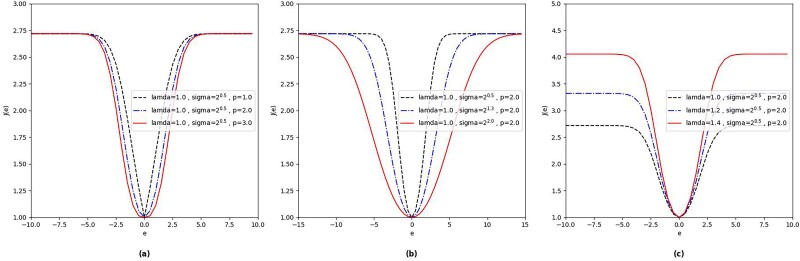
Kernel function curve of KRP with different parameters.

**Figure 3:**
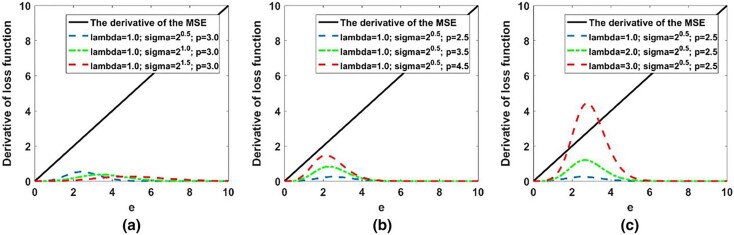
The derivative of KRP with different parameters, as well as the derivative of the MSE [35].

For two different column vector sets with *N* samples each, the higher their similarity, the smaller the value of the corresponding KRP. The target of the model training process is to reduce the KRP value to its lowest, i.e. minimize the discrepancy between the predicted outcome and the desired outcome. Incorporating the KRP function to the KNN model can transform the feature space into a higher-dimensional space where the data can be more easily separable. This can improve the classification performance by allowing the model to capture complex relationships between features. The kernel function of the KRP has upper and lower bounds, which will not lead to an explosion of the function value when the two peptide sequence features are very different from each other. Furthermore, the number of samples is limited; therefore, the KRP expression is approximated using the following formula:


(7) \begin{equation*} {L}_{\lambda, p}\left(A,B\right)=\frac{1}{N\lambda}\sum_{i=1}^N\exp \left\{\lambda{\left[1-{\kappa}_{\sigma}\left({a}_i-{b}_i\right)\right]}^{\frac{p}{2}}\right\} \end{equation*}

where${\left({a}_i,{b}_i\right)}_{i=1}^N$ stands for *N* samples available. Let $A=({a}_1,{a}_2,\dots,\\{a}_N)^T$, $B={\left({b}_1,{b}_2,\dots, {b}_N\right)}^T$*,*$e=a-b$*,*$J(e)=\exp \{\lambda{[1-{\kappa}_{\sigma }(e)]}^{\frac{p}{2}}\}$. Figure 2 shows the kernel function curve of KRP under different parameter constraints. As shown in Fig. 2, the properties of the KRP function include upper and lower boundedness, smoothness without breakpoints, and symmetry in the domain of definition. Figure 3 [35] shows the derivative curve of KRP and the mean square error (MSE). From Fig. 3, the rate of increase of the MSE derivative is directly proportional to the escalation of the error. Conversely, the derivative of the KRP initially rises slightly, then experiences a swift decline, ultimately approaching zero. This behavior implies that KRP mitigates the detrimental impact of outliers and noise, endowing it with an advantage over MSE in terms of robustness.

## Proposed method

### Kernel risk sensitive loss-based k-nearest neighbor model

For HKNN, when an outlier point occurs in the class *c*, it will lead to the corresponding hyperplane having a large deviation from the ideal hyperplane, which will negatively impact the results. Within this study, KRP is introduced into HKNN to significantly mitigate the impact of outliers and noise. Taking each class as a unit, unlike the original HKNN, which sums the distances between each test sample in all test samples and its corresponding local hyperplane of class *c* to directly obtain the KRP objective function value for the entire test dataset, this study forms a KRP objective function for each test sample to achieve a more accurate classification. The summation term is then converted into the sum of the eigenvalues in each dimension of the KNN samples of the test sample.

For class *c*, the smaller the sum of the KRP errors of each feature dimension of the test sample and the sum of the weights of that dimension of its K nearest neighbors in class *c*, the more likely the test sample was to be categorized as class *c*. Furthermore, this study also introduced the Laplace regularization term, which prevented the model from overfitting and improved the model’s generalization ability, and the specific objective function expression is as follows:


(8) \begin{align*} \underset{\alpha_c}{\mathrm{argmin}}{LH}_c(x)=&\ {\lambda}_r{\left\Vert{\alpha}_c\right\Vert}^2+\frac{1}{\lambda d}\sum_{j=1}^d\exp \left\{\lambda{\left[1-{\kappa}_{\sigma}\left({x}_j-{V}_{c,j}{\alpha}_c\right)\right]}^{\frac{p}{2}}\right\}\nonumber\\&+\mu \sum_{f=1}^K\sum_{g=1}^K{W}_{c, fg}{\left({\alpha}_{c,f}-{\alpha}_{c,g}\right)}^2 \end{align*}

where ${V}_c\in{R}^{d\times K}$ is the feature matrix comprising the features of the K closest points of the instance in the *c*th class; ${W}_c\in{R}^{K\times K}$ is the adjacency matrix that indicates whether the K nearest neighbors are K nearest neighbors to each other; if so*,*${W}_{c, fg}$ is computed using similarity matrix computation methods; otherwise ${W}_{c, fg}=0$. The Laplace regularization term of the objective expression is represented using a Laplace matrix combined with Laplace mapping, and the objective function is converted into the following form:


(9) \begin{align*} \underset{\alpha_c}{\mathrm{argmin}} {LH}_c(x)=&\ {\lambda}_r{\left\Vert{\alpha}_c\right\Vert}^2+\frac{1}{\lambda d}\sum_{j=1}^d\exp \left\{\lambda{\left[1-{\kappa}_{\sigma}\left({x}_j-{V}_{c,j}{\alpha}_c\right)\right]}^{\frac{p}{2}}\right\}\nonumber\\&+\mu trace(({\alpha}_{c})^{T}L^{sn}{\alpha}_{c}) \end{align*}

where ${L}^{sn}\in{R}^{K\times K}$ is the normalized Laplace matrix that avoids the problem of excessive or vanishing gradients within the model, ${L}^{sn}$ is calculated as follows:


(10) \begin{equation*} {L}_{ij}^{sn}\left\{\!\!\!\!\!\begin{array}{c}1,\kern9em \mathrm{if}\ i=j\ \mathrm{and}\ {D}_{ii}\ne 0\kern4.45em \\{}-\frac{1}{\sqrt{D_{ii}{D}_{jj}}},\kern7.25em \mathrm{if}\ i\ne j\ \mathrm{and}\ {v}_i\ \mathrm{is}\ \mathrm{adjacent}\ \mathrm{to}\ {v}_j\kern0.5em \\{}0,\kern9em \mathrm{otherwise}\kern8.25em \end{array}\right. \end{equation*}

where ${D}_{ii}=\sum_{j=1}^n{W}_{ij}$, diagonal matrix *D* has its *i*th diagonal element set to the aggregate of the elements in the *i*th row of the matrix *W*.

The objective function attains its lowest value when evaluated at $\frac{\partial \left({LH}_c\left({\alpha}_c\right)\right)}{\partial{\alpha}_c}=0$, which follows:


(11) \begin{align*} 2{\lambda}_r{\alpha}_c-\frac{1}{\lambda d}\sum_{j=1}^d&\left(-\frac{\lambda p}{2{\sigma}^2}\exp \left(\lambda{\left(1-{\kappa}_{\sigma}\left({e}_j\right)\right)}^{\frac{p}{2}}\right)\left({\left(1-{\kappa}_{\sigma}\left({e}_j\right)\right)}^{\frac{p-2}{2}}\right)\right.\nonumber\\& \left.{\kappa}_{\sigma}\left({e}_j\right){V}_{c,j}{e}_j\right)+2\mu{L}^{sn}{\alpha}_c=0 \end{align*}

(12) \begin{equation*} \Rightarrow 2{\lambda}_r{\alpha}_c-\frac{p}{2{\sigma}^2d}{V}_c^T\varLambda \left(X-{V}_c{\alpha}_c\right)+2\mu{L}^{sn}{\alpha}_c=0 \end{equation*}

(13) \begin{equation*} \Rightarrow{\lambda}_1{\alpha}_c-{V}_c^T\varLambda \left(X-{V}_c{\alpha}_c\right)+{\lambda}_2{L}^{sn}{\alpha}_c=0\qquad\quad\, \end{equation*}

(14) \begin{equation*} \Rightarrow{\alpha}_c={\left({\lambda}_1{I}_k+{V}_c^T\varLambda{V}_c+{\lambda}_2{L}^{sn}\right)}^{-1}{V}_c^T\varLambda X\qquad\quad \end{equation*}

where ${\lambda}_1=\frac{4{\sigma}^2d{\lambda}_r}{p}$, ${\lambda}_2=\frac{4{\sigma}^2 d\mu}{p}$, (11) is valid provided when *d > K*. To simplify the representation, the following definition is made:


(15) \begin{equation*} {e}_j={x}_j-{V}_{c,j}{\alpha}_c \end{equation*}

(16) \begin{equation*} {\varLambda}_{jj}=\exp \left\{\lambda{\left[1-{\kappa}_{\sigma}\left({e}_j\right)\right]}^{\frac{p}{2}}\right\}{\left[1-{\kappa}_{\sigma}\left({e}_j\right)\right]}^{\frac{p}{2}}{\kappa}_{\sigma}\left({e}_j\right) \end{equation*}

where $\Lambda$ is a diagonal matrix, ${\Lambda}_{jj}$ is its *j*th diagonal element.

### K-nearest neighbor model based on multi-Laplacian and kernel risk sensitive loss

**Figure 4:**
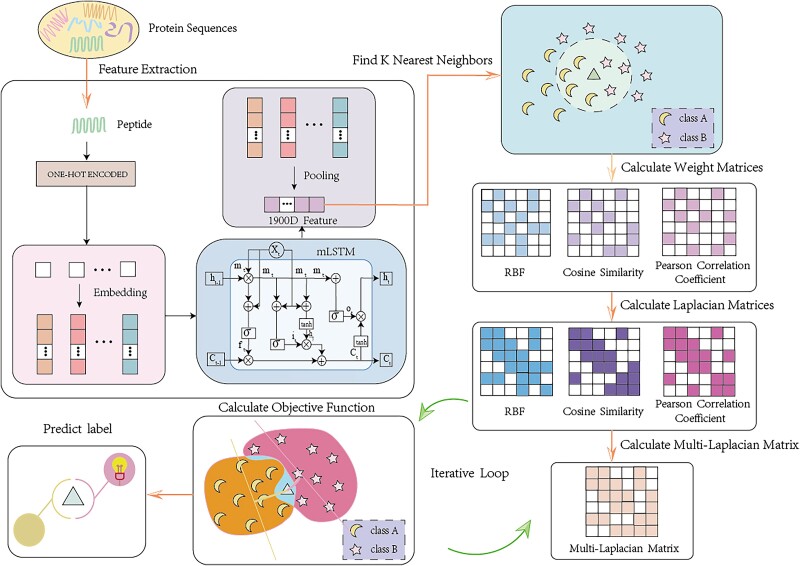
Framework of the proposed approach.

Different matrix similarity calculation methods can obtain different Laplace matrices representing different graph signal features. Laplacian regularization can make full use of the spatial structure information of the data, penalize the parameters through the Laplacian matrix, and make the model smoother. The multi-Laplacian matrix obtained by integrating multiple similarity calculation methods can retain the connection between the data from different perspectives, further reducing the model’s sensitivity to the local noise sensitivity of the model. Even if there is noise or error in one of the matrices, information from the other matrices can still provide valid features, thereby reducing dependence on a single matrix. Figure 4 presents the framework of the methodology put forward.

In the research, three similarity matrix calculations were used to compute three different Laplace matrices for fusion.


(17) \begin{equation*} {W}_{ij}=\exp \left(-\gamma{\left\Vert{x}_i-{x}_j\right\Vert}^2\right)\qquad\qquad\quad\ \,\qquad \end{equation*}

(18) \begin{equation*} {W}_{ij}=\frac{x_i\bullet{x}_j}{\left\Vert{x}_i\right\Vert \times \left\Vert{x}_j\right\Vert }\qquad\qquad\qquad\qquad\quad\qquad \end{equation*}

(19) \begin{equation*} {W}_{ij}=\frac{\sum_{a=1}^d\left({x}_{ia}-\overline{x_i}\right)\left({x}_{ja}-\overline{x_j}\right)}{\sqrt{\sum_{a=1}^d{\left({x}_{ia}-\overline{x_i}\right)}^2}\sqrt{\sum_{a=1}^d{\left({x}_{ja}-\overline{x_j}\right)}^2}} \end{equation*}

where (17) is the RBF function, (18) is the cosine similarity formula, and (19) is the Pearson correlation coefficient formula. The normalized Laplace matrix can be obtained using (10). The integrated Laplace matrix is expressed as follows [37]:


(20) \begin{equation*} {L}_d^{\ast }=\sum_{v=1}^m{\left({\beta}_v\right)}^{\rho }{L}_v^{sn},\kern0.5em s.t.\sum_{v=1}^m{\beta}_v=1,{\beta}_v\in \left[0,1\right] \end{equation*}

where ${L}_d^{\ast }$ is the fused multi-Laplacian matrix, ${L}_v^{sn}$ is the *v*th normalized Laplacian matrix, *m* is the number of kinds of Laplacian matrix computation methods, and ${\beta}_v$ is the weight of the *v*th normalized Laplacian matrix. The multi-Laplace representation is modified to the following form:


(21) \begin{equation*} {\displaystyle \begin{array}{c}\mathrm{argmin}\ \\{}{\beta}_v\end{array}}\mathrm{trace}\left({\alpha}_c^T\left(\sum_{v=1}^m{\left({\beta}_v\right)}^{\rho }{L}_v^{sn}\right){\alpha}_c\right)\kern1em \mathrm{s}.\mathrm{t}.\sum_{v=1}^m{\beta}_v=1,{\beta}_v\in \left[0,1\right]. \end{equation*}

The initial setting of ${\mathrm{\beta}}_v$ is $\frac{1}{m}$. The Laplace multiplier method was used to solve the optimization objective by combining the constraints and functions. The corresponding Laplace function is expressed as:


(22) \begin{equation*} L\left({\beta}_v,\varepsilon \right)=\mathrm{trace}\left({\alpha}_c^T\left(\sum_{v=1}^m{\left({\beta}_v\right)}^{\rho }{L}_v^{sn}\right){\alpha}_c\right)-\varepsilon \left(\sum_{v=1}^m{\beta}_v-1\right). \end{equation*}

The key equation in the KKT condition combined with the end condition for the partial derivatives to be zero is:


(23) \begin{equation*} \left\{\!\!\begin{array}{c}\rho{\left({\beta}_v\right)}^{\left(\rho -1\right)}\mathrm{trace}\left({\alpha}_c^T{L}_v^{sn}{\alpha}_c\right)-\varepsilon =0\\[5pt] {}\sum_{v=1}^m{\beta}_v-1=0\end{array}\right.. \end{equation*}

The final representation of $ {\mathrm{\beta}}_v $ is:


(24) \begin{equation*} {\beta}_v=\frac{tm}{\sum_{v=1}^m tm}\qquad\ \ \ \quad\qquad \end{equation*}

(25) \begin{equation*} tm={\left(\frac{1}{\mathrm{trace}\left({\mathrm{\alpha}}_{\mathrm{c}}^{\mathrm{T}}{L}_v^{sn}{\alpha}_c\right)}\right)}^{\frac{1}{\rho -1}}. \end{equation*}

The objective function of the classifier with the multi-Laplacian regularization term is shown below, and the hyperparameter ${\mathrm{\alpha}}_c$ in the objective function can be obtained by combining the obtained ${\mathrm{\beta}}_v$ with (20) to obtain the latest integrated normalized multi-Laplacian matrix, following substitution into (14) yields, and then replacing ${L}^{sn}$ in (9) with ${L}_d^{\ast }$ to obtain the final function:


(26) \begin{align*} \underset{\alpha_c}{\mathrm{argmin}}{LH}_c(x)=&\ {\lambda}_r{\left\Vert{\alpha}_c\right\Vert}^2+\frac{1}{\lambda d}\sum_{j=1}^d\exp \left\{\lambda{\left[1-{\kappa}_{\sigma}\left({x}_j-{V}_{c,j}{\alpha}_c\right)\right]}^{\frac{p}{2}}\right\}\nonumber\\&+\mu trace(({\alpha}_{c})^{T}L^{*}_{d}{\alpha}_{c}) \end{align*}

The specific algorithmic process is shown in Table 1.

**Table 1:** Algorithm of K-nearest neighbor model based on multi-Laplacian and kernel risk sensitive loss

**Algorithm of K-Nearest Neighbor Model based on Multi-Laplacian and Kernel Risk Sensitive Loss**
**Input: data set** $\left\{X,Y\right\}={\left\{{x}_i,{y}_i\right\}}_{i=1}^N$ ; parameter set $\left\{{\lambda}_r,\lambda, \sigma, p,\rho, \mu, \gamma \right\}$; Feature dimension d (can be obtained from the size of the feature matrix); K (the quantity of neighboring data points); iterations (iteration count); m (the tally of weight matrix categories)
**Steps:**
**Step 1:** Separate the dataset into a training set and a test set
**Step 2:** for test_data = $\left\{x\_{test}_i,y\_{test}_i\right\},i=0,1,\dots, N$
Find the K samples within the training set of class c that are closest to this test sample i based on the Euclidean distance to compute ${V}_c,c\in \left\{0,1\right\}$
**Step 3:** Random initialize ${\alpha}_{c,0}$, initialize ${\beta}_v$ with the mean value $\frac{1}{m}$
**Step 4:** Calculate the different weight matrices to get the normalized Laplacian matrix ${L}_v^{sn}$
**Step 5:** for $\#\mathrm{iter}=1,2,\dots, \mathrm{iterations}$
Compute the normalized Multi-Laplacian matrix ${L}_d^{\ast }$
Compute the diagonal matrix Λ Update ${\alpha}_{c, iter}$
Compute ${\beta}_v$
**Step 6:** Calculate the objective function ${LH}_c,y\_{pred}_i=c\_\mathit{\min},{LH}_{c\_\mathit{\min}}=\mathit{\min}\left({LH}_c\right)$
**Output:** the predict label of x_test

## Results and discussion

### Kernel risk sensitive loss-based k-nearest neighbor model

**Figure 5:**
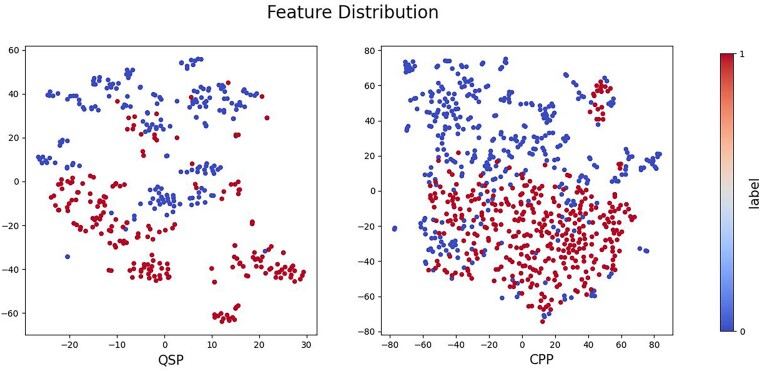
Feature distribution.

The therapeutic peptide datasets were derived from the QSP collection made available by Wynendaele *et al.* [38] and Rajput *et al.* [39], and the CPP dataset provided by Guo *et al.* [40]. The distribution of the features in the dataset is shown in Fig. 5. Among them, QSP dataset includes 440 samples, and CPP dataset includes 924 samples. The number of negative and positive samples in both datasets was equivalent. Specific dataset information is presented in Table 2.

### Performance evaluation indicators

The research assessed the model’s performance utilizing the accuracy (ACC), sensitivity (SN), specificity (SP), and Matthew correlation coefficient (MCC) as metrics [41–43].


(27) \begin{equation*} \mathrm{ACC}=\frac{\mathrm{TP}+\mathrm{TN}}{\mathrm{TP}+\mathrm{TN}+\mathrm{FP}+\mathrm{FN}} \end{equation*}

(28) \begin{equation*} \mathrm{SN}=\frac{\mathrm{TP}}{\mathrm{TP}+\mathrm{FN}} \end{equation*}

(29) \begin{equation*} \mathrm{SP}=\frac{\mathrm{TN}}{\mathrm{TN}+\mathrm{FP}} \end{equation*}

(30) \begin{equation*} \mathrm{MCC}=\frac{\mathrm{TP}\times \mathrm{TN}-\mathrm{FP}\times \mathrm{FN}}{\sqrt{\left(\mathrm{TP}+\mathrm{FN}\right)\times \left(\mathrm{TP}+\mathrm{FP}\right)\times \left(\mathrm{TN}+\mathrm{FN}\right)\times \left(\mathrm{TN}+\mathrm{FP}\right)}} \end{equation*}

ACC denotes the ratio of accurately classified instances out of the total instances, SN signifies the fraction of true positive predictions within the positive instances, and SP indicates the fraction of true negative predictions within the negative instances. MCC can comprehensively consider four classification results, handle imbalanced datasets, and summarize the classification quality.

**Table 2:** The training and test set information of the dataset

Datasets	Training set		Test set	
	Positive	Negative	Positive	Negative
QSP	200	200	20	20
CPP	370	370	92	92

### The ablation experiments

To validate the introduction of the HKNN algorithm, KRP and multiple Laplacian matrices can improve the classifier performance, relevant ablation experiments were carried out. A five-fold cross validation of the training set on both datasets verified that the proposed classifier outperformed the subclassifiers with some modules removed. Among them, the KNN model is based on multi-Laplacian and kernel risk-sensitive loss, i.e. MvHKNN(KRP) on the QSP (ACC = 0.935, SN = 0.930, SP = 0.944, MCC = 0.870) and CPP datasets (ACC = 0.901, SN = 0.903, SP = 0.900, MCC = 0.804).

All experiments were conducted on an Intel(R) Core(TM) i7-10,710 U (1.61 GHz) CPU and Python 3.10.2. The results showed that MvHKNN(KRP) has a better generalization performance. With the introduction of the HKNN algorithm, KRP, and multi-Laplacian matrices, the accuracy of the CPP dataset continuously improved, indicating that all three modules contributed to the performance improvement of the classifier. Laplacian regularization involves a penalty on the second derivative of the function, promoting solutions that are smooth across the input space. The spatial structural information is considered, the performance is improved by better preserving the structured information through the introduction of the adjacency matrix component, and the generalization of the model to new, unseen data is improved. Therapeutic peptide data contain high-dimensional features that have a high risk of overfitting, but the Laplace regularization term contains squared terms, which generate smoother decision boundaries during the training process and effectively avoid overfitting. The results of the ablation experiments are listed in Tables 3 and 4.

**Table 3:** Outcomes of various KNN-inspired frameworks on the QSP training dataset

Framework	ACC	SN	SP	MCC
KNN	87.8	80.0	82.8	76.5
HKNN	93.0	92.5	92.8	86.1
HKNN(KRP)	92.3	92.5	92.5	84.5
MvHKNN(KRP)	**93.5**	**93.0**	**94.4**	**87.0**

**Table 4:** Outcomes of various KNN-inspired frameworks on the CPP training dataset

Framework	ACC	SN	SP	MCC
KNN	82.4	75.9	72.5	67.4
HKNN	88.0	83.8	85.1	76.3
HKNN(KRP)	89.1	88.9	89.0	78.2
MvHKNN(KRP)	**90.1**	**90.3**	**90.0**	**80.4**

### Computational complexity analysis

The computational cost of the HKNN algorithm is mainly spent on calculating the distances between the samples to be classified and the K nearest neighbors in the same class, which leads to a time complexity of $O(MNd)$, where *M* is the number of training samples, *d* is the feature dimension, and *N* is the number of samples to be classified. The KRP algorithm’s computational cost is mainly spent on calculating the matrix Λ and updating the weight matrices in each round of iteration. The time complexity of computing the matrix Λ is $O(dK)$ and the time complexity of computing the weight matrix ${\alpha}_c$ is $O\left(d{K}^2\right)$, where *K* is the number of nearest neighbors. Finally, the time complexity of the KRP algorithm in each round of iterations is $O\left(d{K}^2\right)$, and ultimately, the time complexity of the entire KRP algorithm is $O\left(\mathrm{iteration}\bullet Nd{K}^2\right)$. The MvHKNN(KRP) algorithm adds to the HKNN and KRP algorithms the time to compute the multi-Laplacian matrix at each iteration, and the time complexity of this step is $O\left(N{K}^3\right)$, since the actual dataset $d>K$, the time complexity of the algorithm is essentially the same as that of KRP. As shown in Table 5, the KRP takes longer than the HKNN and the KNN because, in addition to the time cost of calculating the distances, the computational cost due to the increase in the complexity of the model is also spent. With the addition of the multi-Laplace matrix regularization, the complexity of the model increases due to the need to compute multiple Laplace matrices, making the algorithm even more computationally intensive. In addition, HKNN and KNN do not have a loop iteration process, so the time cost will be lower than the KRP and MvHKNN(KRP), but the iterative loops make the model more robust, the iterative loops of the KRP make the model more resistant to noise and outliers, and the multi-Laplacian regularization makes the model more generalizable.

**Table 5:** CPU time of KNN-based models

Framework	Runtime on QSP training set	Runtime on QSP independent set	Runtime on CPP training set	Runtime on CPP independent set
KNN	94.5682	15.9244	375.5314	149.5777
HKNN	98.8653	20.2991	395.4709	155.8550
HKNN(KRP)	274.0981	29.6999	567.0560	187.4899
MvHKNN(KRP)	402.9725	32.6517	568.2443	188.1022

**Figure 6:**
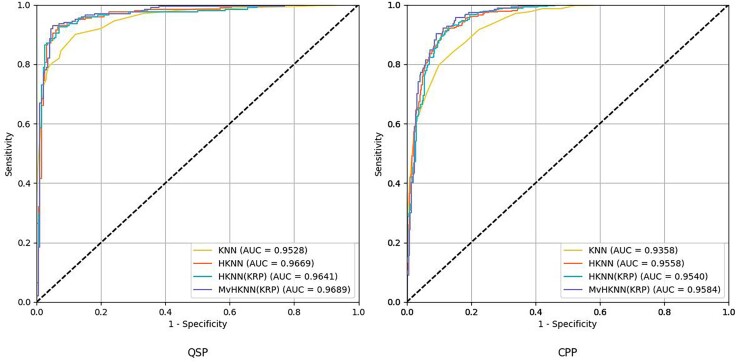
The ROC curves and AUC values of various frameworks on training datasets.

### Comparison experiment with existing frameworks on training datasets

To demonstrate the competitiveness of the proposed model in predicting therapeutic peptides, we compared PPTPP [44], ITP-Pred [45], SVM, LSTM, TSK-FS [40], KRP-ESN [33], and ESN-based models [46] with the MvHKNN(KRP) on the relevant dataset. Figure 6 illustrates the ROC curves and the corresponding AUC metrics for the assorted KNN-derived models applied to the training datasets. The results show that the MvHKNN(KRP) has the best performance. Tables 6 and 7 present the outcomes of the five-fold cross-validation process for the above models on the QSP and CPP training sets, respectively. As shown in Table 6, the MvHKNN(KRP) achieved optimal results, with 3.7% improvement in accuracy, 3.1% improvement in sensitivity, 4.8% improvement in specificity, and 7.4% improvement in MCC compared with the WESN-SP [SBL] algorithm. The MvHKNN(KRP) is 6.8% higher than TSK-FS [40] and 5.4% higher than KRP-ESN [33] in ACC. This demonstrates that the MvHKNN(KRP) has a higher prediction accuracy for both negative and positive samples. As shown in Table 7, the MvHKNN(KRP) achieved optimal results regarding both ACC and MCC, with 0.2% improvement in accuracy and 0.5% improvement in MCC compared to WESN-SP (SBL), which had a superior ability to deal with imbalanced negative–positive samples and a better prediction of the accuracy of therapeutic peptides. The MvHKNN(KRP) is 0.3% higher than TSK-FS [40] and 3.7% higher than KRP-ESN [33] in ACC. As shown in Table 8, on the QSP training set, the *P*-value between MvHKNN(KRP) and the best result, WESN-SP(SBL), is 0.0017. On the CPP training set, the *P*-value between MvHKNN(KRP) and WESN-SP(SBL) is 0.0377, and although the accuracy, MvHKNN(KRP), is only 0.2% higher than WESN-SP(SBL) by 0.2%, but the *P*-value between the two is <0.05, indicating that there is a significant difference between the two methods in CPP prediction and that the 0.2% increase in accuracy brings about a further improvement in performance. Multi-Laplacian regularization method and square penalty value are incorporated to penalize large weights in the model, reducing the complexity and the risk of overfitting. Besides, five-fold cross-validation was introduced to help in assessing the model's performance on different subsets of the data, ensuring that the model does not overfit to a specific subset. Moreover, in the iterative algorithm for objective function computation, due to the fast convergence of machine learning, the experiment is iterated in the third round, and the training is stopped in time to prevent model overfitting before the model has a chance to fit the noise in the training data.

**Table 6:** Outcomes of various frameworks on the QSP training dataset

Framework	ACC	SN	SP	MCC
KNN	87.8	80.0	82.8	76.5
HKNN	93.0	92.5	92.8	86.1
HKNN(KRP)	92.3	92.5	92.5	84.5
MvHKNN(KRP)	**93.5**	**93.0**	**94.4**	**87.0**
WESN-SP(SBL) [46]	89.8	89.9	89.6	79.6
PPTPP [44]	83.5	85.5	85.0	70.5
SVM [45]	66.0	63.3	76.0	32.1
LSTM [45]	56.5	67.8	27.0	16.8
ITP-Pred [45]	87.0	85.5	88.0	72.9
TSK-FS [40]	86.7	88.8	84.3	73.4
KRP-ESN [33]	88.1	86.1	89.5	76.0

**Table 7:** Outcomes of various frameworks on the CPP training dataset

Framework	ACC	SN	SP	MCC
KNN	82.4	75.9	72.5	67.4
HKNN	88.0	83.8	85.1	76.3
HKNN(KRP)	89.1	88.9	89.0	78.2
MvHKNN(KRP)	**90.1**	90.3	90.0	**80.4**
WESN-SP(SBL) [46]	89.9	**91.8**	88.0	79.9
PPTPP [44]	74.9	71.6	78.1	49.8
SVM [45]	76.6	71.7	88.4	64.9
LSTM [45]	80.0	83.2	77.6	61.7
ITP-Pred [45]	89.0	86.3	**93.2**	78.7
TSK-FS [40]	89.8	90.6	89.0	79.6
KRP-ESN [33]	86.4	89.8	83.3	73.1

### Comparison experiments with existing models on independent testing set

**Figure 7:**
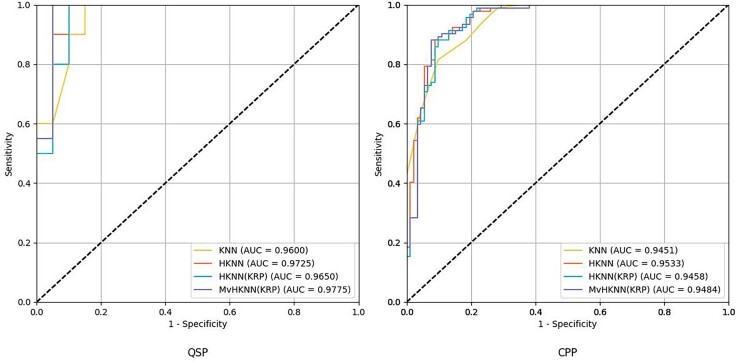
The ROC curves and AUC values of various frameworks on an independent testing set.

**Figure 8:**
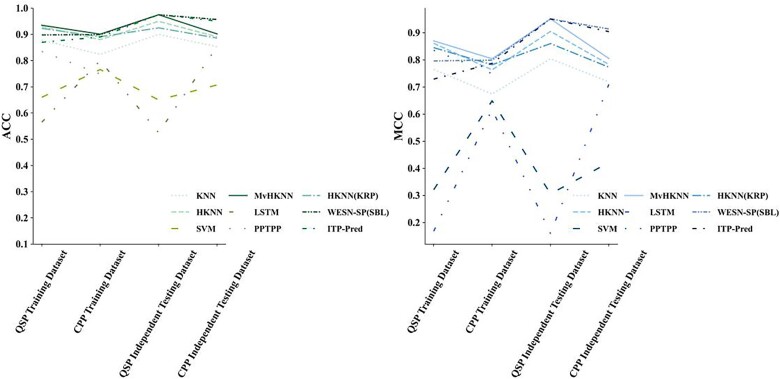
ACC and MCC of different models on the training datasets and independent datasets.

In this study, experiments were conducted on the QSP and CPP independent test sets, and the findings are shown in Tables 9 and 10. Observing Table 9, the MvHKNN(KRP) achieves comparable results to WESN-SP (SBL) and is slightly inferior to ITP-Pred in terms of sensitivity. The MvHKNN(KRP) is 2.5% higher than TSK-FS [40] and 7.5% higher than KRP-ESN [33] in ACC. Figure 7 depicts the ROC curves and AUC values of various KNN-based models on the independent testing set, where the MvHKNN(KRP) has the best performance. In Table 10, MvHKNN(KRP) is 4.9% lower than TSK-FS [40] and 4.9% higher than KRP-ESN [33] in ACC. The proposed model is not as effective as WESN-SP (SBL) regarding the assessment criteria. The rationale for the poor results of the CPP-independent test set may be that, first, the data distribution of CPP in the independent test dataset may be significantly different from that in the training set, whereas the composition of QSP peptides might exhibit a greater degree of similarity across both training and test sets. Second, the CPP dataset contains more noise and outliers and suffers from a greater data-quality problem. Furthermore, according to Table 11, the CPP dataset has very similar peptide sequence lengths for the positive and negative samples; the positive and negative samples may overlap more in the feature space while masking the actual boundaries between the different categories, which makes it difficult for the classifier to distinguish between them. Contrary, the predicted values of QSP positive samples are more concentrated and far away from the predicted values of negative samples, which can be better learned by the proposed method with the corresponding feature distributions. More interestingly, the predicted values of negative samples in the QSP dataset are more broadly distributed, which would have made distinguishing the negative samples more challenging, but it also demonstrates that the proposed method can still hold the excellent prediction performance when facing the inhomogeneous length samples and is more susceptible to the interference of sample inhomogeneity. The ACC and MCC performance of different models on the QSP training set and the independent test set and CPP training set and the independent test set are shown in Fig. 8.

**Table 8:** Outcomes of *t*-test for the proposed model and other models

Framework	QSP	CPP
WESN-SP(SBL) [46]	0.0017	0.0377
PPTPP [44]	0.0003	<1e-4
SVM [45]	<1e-4	<1e-4
LSTM [45]	<1e-4	<1e-4
ITP-Pred [45]	0.0005	0.0010
TSK-FS [40]	0.0005	0.0152
KRP-ESN [33]	0.0008	<1e-4

**Table 9:** Outcomes of various frameworks on the QSP independent testing dataset

Framework	ACC	SN	SP	MCC
KNN	90.0	95.0	85.0	80.4
HKNN	95.0	100.0	90.0	90.5
HKNN(KRP)	92.5	**100.0**	85.0	86.0
MvHKNN(KRP)	**97.5**	**100.0**	95.0	**95.1**
WESN-SP(SBL) [46]	**97.5**	**100.0**	95.0	**95.1**
PPTPP [44]	–	–	–	–
SVM [45]	65.0	62.5	75.0	30.6
LSTM [45]	52.5	1.0	5.0	16.0
ITP-Pred [45]	97.5	95.2	**100.0**	95.1
TSK-FS [40]	95.0	100.0	90.0	90.4
KRP-ESN [33]	90.0	95.0	85.0	80.4

**Table 10:** Outcomes of various frameworks on the CPP independent testing dataset

Framework	ACC	SN	SP	MCC
KNN	85.3	94.6	76.1	71.9
HKNN	89.1	88.0	90.2	78.3
HKNN(KRP)	88.6	85.9	91.3	77.3
MvHKNN(KRP)	90.2	88.0	92.4	80.5
WESN-SP(SBL) [46]	**95.7**	**97.8**	93.5	**91.4**
PPTPP [44]	-	-	-	-
SVM [45]	70.7	62.7	80.4	42.1
LSTM [45]	85.3	89.2	80.4	71.0
ITP-Pred [45]	95.1	92.8	**97.8**	90.4
TSK-FS [40]	95.1	95.6	94.5	90.2
KRP-ESN [33]	87.0	89.1	84.8	74.0

**Table 11:** The summary of statistic (sequence length) for QSP and CPP

Datasets	Statistic	QSP	CPP
Positive	Mean Std.	11.26 6.42	20.22 8.39
Negative	Mean Std.	33.0 17.30	20.48 8.41

## Conclusions

To better solve the problem of therapeutic peptide prediction, this study proposes a k-nearest neighbor model based on multi-Laplacian and kernel risk-sensitive loss, where the loss function is a KRP loss function using a Gaussian kernel. The model selects the k closest neighbors in each class for every test instance to construct a hyperplane and classifies the test samples according to the distance between the hyperplanes of different classes and test samples. The results in Tables 3 and 4 also show that the model with the addition of the HKNN algorithm shows a significant increase in all four metrics compared to the KNN model. Simultaneously, the classifier does not treat all test samples as a whole and only uses one objective function, but rather ‘customizes’ the objective function in different categories for each test sample. Besides, the model improves on the original KRP function, which accumulates the loss values of all samples into an overall objective function. Instead of summing the weights of different test samples within a Gaussian kernel, the weights of different dimensions of one sample are summed within a Gaussian kernel. The objective function obtained in this manner is specific to a test sample, which enhances the framework’s capacity to generalize. From Table 4, it can be seen that the accuracy of the model is further improved with the addition of the KRP loss function, which indicates that the use of the KRP loss function improves the predictive ability of the model. In addition, the system utilizes several similarity methods to obtain different Laplace matrices to better retain and utilize the connections between different samples, reduce the interference of anomalous and noisy samples in the construction of the objective function, and increase the robustness of the system. From Tables 3 and 4, it can be seen that the addition of the multi-Laplacian regularization gives better results in the evaluation metrics of the model, which indicates that multi-Laplacian is an effective regularization method to improve the prediction performance.

The MvHKNN(KRP) achieved superior performance over other classifiers in the training set five-fold cross-validation experiments, and also performed well on the QSP independent test set; taken together, the MvHKNN(KRP) is an effective classifier for predicting proteins. However, the competence of the model on the CPP independent test dataset was not satisfactory. The performance on the CPP independent test set reflects certain limitations of the study. The feature extraction fails to adequately capture the key characteristics of the CPP dataset, and some of the selected features may not be discriminative or even intrusive in the CPP dataset, which makes the model ineffective in reflecting the true differences between the positive and negative samples. The experiment lacks diversity of dataset types, and the selected datasets also belong to small datasets. On the one hand, inconsistent feature distributions and differences in the data size of QSP and CPP may lead to variations in model prediction performance. On the other hand, whether the model is suitable for predicting other types of therapeutic peptides is yet to be verified, and the performance on the current dataset may not fully generalize to other datasets. The model proposed in this study is constructed based on the KNN model, which performs well when dealing with small datasets, but the computational complexity of KNN increases dramatically with the increase of data volume or the complexity of features, resulting in a significant expansion of the computational cost.

Future work will consist of performing feature engineering to improve existing feature extraction methods. In addition to the UniRep feature extraction used in the paper, other feature engineering can be considered to be incorporated to capture missing sequence information, including manual extraction of features like physicochemical properties. Other deep learning models can be utilized to extract peptide sequence features from evolutionary information and structural information and structural information [47]. Simultaneously, the stability of the model requires improvement. We consider using integrated learning methods, such as random forests or gradient-boosted trees, to combine predictions from multiple models. More robust algorithms, such as heuristic algorithms [48] and supervised learning algorithms [49], are combined in model fusion. In addition, applying data augmentation techniques to the model, such as SMOTE or ADASYN, which increases the number of samples from a few classes, can improve the balance of the model and increase its robustness. Optimization methods can improve the predictive performance of the model by finding the optimal combination of hyperparameters using methods, such as grid search, stochastic search, or Bayesian optimization. Adding a penalty component to the loss formula in combination with other regularization terms [50] can mitigate the effect of anomalous and noisy samples on the model. In addition, neural networks have stronger learning capabilities [51], which can stack deep learning techniques on top of machine learning, and simplify neural network models by combining discard [52] and attention mechanisms [53] to obtain higher prediction accuracy and robustness.

**Key Points**

- The KRP was introduced into the HKNN model as a loss function that supersedes the original squared loss function. An objective function was constructed for each sample within the classes targeted for classification, aiming to enhance the model’s generalization capabilities.
- Laplace mapping and regularization were introduced, combining multiple Laplace matrices, using multiview learning to fully utilize the scores of different similarity methods, and employing a repetitive computational process to address the objective function.
- The peptide sequences obtained from the pre-training of the embedding model using deep representation learning were used as feature vectors to better preserve the key information of the peptide sequences.
- The proposed model achieves good performance in predicting therapeutic peptides.

## Data Availability

The dataset comes from the work of Guo [46] et al. If the implementation of our proposed method is needed, please send an email to the author.
